# Multi-gene phylogenetic evidence suggests *Dictyoarthrinium* belongs in Didymosphaeriaceae (Pleosporales, Dothideomycetes) and *Dictyoarthrinium musae* sp. nov. on *Musa* from Thailand

**DOI:** 10.3897/mycokeys.71.55493

**Published:** 2020-08-05

**Authors:** Binu C. Samarakoon, Dhanushka N. Wanasinghe, Milan C. Samarakoon, Rungtiwa Phookamsak, Eric H. C. McKenzie, Putarak Chomnunti, Kevin D. Hyde, Saisamorn Lumyong, Samantha C. Karunarathna

**Affiliations:** 1 Department of Biology, Faculty of Science, Chiang Mai University, Chiang Mai 50200, Thailand; 2 Center of Excellence in Fungal Research, Mae Fah Luang University, Chiang Rai 57100, Thailand; 3 School of Science, Mae Fah Luang University, Chiang Rai 57100, Thailand; 4 CAS Key Laboratory for Plant Biodiversity and Biogeography of East Asia (KLPB), Kunming Institute of Botany, Chinese Academy of Sciences, Kunming 650201, Yunnan, China; 5 World Agro Forestry Centre, East and Central Asia, 132 Lanhei Road, Kunming 650201, Yunnan, China; 6 Centre for Mountain Futures (CMF), Kunming Institute of Botany, Kunming 650201, Yunnan, China; 7 Innovative Institute of Plant Health, Zhongkai University of Agriculture and Engineering, Guangdong Province, People’s Republic of China; 8 Manaaki Whenua-Landcare Research, Private Bag 92170, Auckland, New Zealand; 9 Research Center of Microbial Diversity and Sustainable Utilization, Faculty of Sciences, Chiang Mai University, Chiang Mai 50200, Thailand; 10 Academy of Science, The Royal Society of Thailand, Bangkok 10300, Thailand

**Keywords:** Banana, *Dictyoarthrinium sacchari*, DNA sequences, Musaceae, one new species, saprobes, taxonomy

## Abstract

Dead leaves of *Musa* sp. (banana) were collected in northern Thailand during an investigation of saprobic fungi. Preliminary morphological observations revealed that three specimens belong to *Dictyoarthrinium*. Phylogenetic analyses of combined SSU, LSU, ITS and *tef*1-α sequence data revealed that *Dictyoarthrinium* forms a clade in Didymosphaeriaceae (Massarineae, Pleosporales, Dothideomycetes) sister to *Spegazzinia*. Based on contrasting morphological features with the extant taxa of *Dictyoarthrinium*, coupled with the multigene analyses, *Dictyoarthrinium musae* sp. nov. is introduced herein. Our study provides the first detailed molecular investigation for *Dictyoarthrinium* and supports its placement in Didymosphaeriaceae (Massarineae, Pleosporales, Dothideomycetes). Previously, *Dictyoarthrinium* was classified in Apiosporaceae (Xylariales, Sordariomycetes).

## Introduction

Hughes (1953) documented seven hyphomycete genera (*Arthrinium*, *Catenospegazzinia*, *Cordella*, *Dictyoarthrinium*, *Endocalyx*, *Pteroconium* and *Spegazzinia*) that had unique basauxic conidiogenous cell development. Hyde et al. (1998) accommodated *Dictyoarthrinium*, *Endocalyx*, *Scyphospora* (= *Arthrinium*) and *Spegazzinia* in Apiosporaceae (Xylariales, Sordariomycetes), based on morphological characteristics. Based on molecular phylogenetic data (LSU and ITS), *Cordella* and *Pteroconium* were synonymised under *Arthrinium* by Crous and Groenewald (2013) and *Arthrinium* was confirmed as the asexual morph of *Apiospora*. With the availability of molecular data (SSU, LSU, ITS and *tef*1-α), Tanaka et al. (2015) transferred *Spegazzinia* to Didymosphaeriaceae. Wijayawardene et al. (2018) and Hyde et al. (2020) accommodated *Arthrinium*, *Dictyoarthrinium* and *Endocalyx*, all with basauxic conidiogenous cell development, in Apiosporaceae.

*Dictyoarthrinium* was introduced by Hughes (1952) with *D. quadratum* as the type species. *Dictyoarthrinium africanum* was simultaneously introduced. Damon (1953) re-examined the type material, descriptions and illustrations of *Tetracoccosporium sacchari* (Johnston and Stevenson 1917) and mentioned that *T. sacchari* was congeneric with *Dictyoarthrinium quadratum*. Therefore, Damon (1953) combined *T. sacchari* as *Dictyoarthrinium sacchari*. Damon (1953) also named *D. quadratum* as the heterotypic synonym of *D. sacchari*. Rao and Rao (1964) introduced *D. lilliputeum* and *D. microsporum*, while Kobayasi et al. (1971) introduced *D. rabaulense* as novel taxa to the genus. Somrithipol (2007) introduced *D. synnematicum* and currently seven epithets of *Dictyoarthrinium* are listed in Index Fungorum (2020). All *Dictyoarthrinium* species were introduced, based only on morphological data. Vu et al. (2019) sequenced *D. sacchari* (CBS 529.73) and submitted LSU data to GenBank as the only valid molecular record for the genus.

*Dictyoarthrinium* is characterised by basauxic conidiogenous cell development (Hughes 1952; Damon 1953; Matsushima 1971). Basauxic development is demonstrated by conidiogenous cells in which elongation occurs at a basal growing point after formation of a single, terminal blastic conidium at its apex (Cole 1976). Conidiophores of *Dictyoarthrinium* are minutely verruculose, subhyaline and transversely septate (Ellis 1971). Usually, the septa are dark brown and appear as thick stripes on the conidiophore. Conidiophore mother cells are often hyaline or pale brown and cup-shaped (Hughes 1952) or subspherical (Ellis 1971). The length of conidiophores varies within the genus, but in some species, the dimensions are more or less similar. Conidia of *Dictyoarthrinium* arise from the conidiophore at terminal or lateral parts. Conidiogenesis is monoblastic or polyblastic and integrated (Ellis 1971). Conidia are simple, solitary, dematiaceous and often four-celled. Some taxa (e.g. *D. africanum*) have 16-celled conidia (Hughes 1952). The surface of conidia is verruculose and most species have warts on the surface. However, the conidia of *D. rabaulense* are densely echinulate with long spines (Kobayasi 1971). The conidia vary in shape from square to spherical, subspherical or oblong. Most conidia appear flattened on one side. As a specific feature, only *D. synnematicum* possesses synnemata with filaments (Somrithipol 2007). Stroma, setae and hyphopodia have not been observed in *Dictyoarthrinium*.

Many *Dictyoarthrinium* species are saprobes that colonise dead plant materials, although *D. rabaulense* was recorded even from soil and air (Kobayasi et al. 1971; Ellis 1976). Most *Dictyoarthrinium* species occur on monocotyledonous plants. The genus is widely distributed across the tropics, mainly in terrestrial environments (Ellis 1971; 1976). The sexual morph of *Dictyoarthrinium* is unknown. Hosts, substrates and geographical distributions of extant *Dictyoarthrinium* species are listed in Table 1.

**Table 1.** Hosts, substrates and geographical distribution of *Dictyoarthrinium* species.

Species	Hosts/substrates	Geographical distribution	References
* Dictyoarthrinium africanum* S. Hughes	* Miscanthus *, *Panicum*, *Paspalum virgatum*, *Saccharum*, leaf litter of *Typha latifolia*	Argentina, Ghana, Solomon Islands, Venezuela	Hughes (1952); Ellis (1971); McKenzie and Jackson (1986); Urtiaga (1986); Tarda et al. (2019)
* D. lilliputeum* P. Rag. Rao and D. Rao	Leaf litter of *Bambusa*	India	Rao and Rao (1964); Sushma et al. (2020)
* D. microsporum* P. Rag. Rao and D. Rao	Dead leaves of *Borassus flabellifer*	India	Rao and Rao (1964)
* D. rabaulense* Matsush.	* Brassica campestris*, *Dendrocalamus strictus*, *Gossypium*, *Xylia xylocarpa*, air and soil	Bismarck Archipelago, Britain, Congo, India, New Caledonia, Nigeria, Tanzania.	Kobayasi et al. (1971); Ellis (1976); Bhat (2010)
* D. sacchari* (J.A. Stev.) Damon = *D. quadratum* S. Hughes	Dead stems and leaves of *Ananas*, *Bambusa*, *Borassus*, *Cassia*, *Cosmos bipinnatus*, *Cymbopogon*, *Delonix elata*, *Dracaena*, *Erythrina*, *Lithachne pauciflora*, *Musa acuminata*, *M. paradisiaca*, *Neolitsea scrobiculata*, *Pandanus*, *Persea mechrantha*, *Phragmites*, *Prunus amygdalus*, *Saccharum* sp., *S. officinarum*, *S. spontanium*, *Zinnia*, leaf litter of *Typha latifolia*, decaying plant materials of dicots	Brazil, Cuba, Federated Ghana, India, Malaysia, Pakistan, Puerto Rico, Solomon Islands, Spain, States of Micronesia, Thailand, Venezuela, Zambia	Hughes (1952); Subramanian (1952); Nair and Tyagi (1961); Srivastava et al. (1964); Dennis (1970); Ellis (1971); Matsushima (1971); Stevenson (1975); Srivastava and Gupta (1981); Arnold (1986); McKenzie and Jackson (1986); Paul and Singh (1986); Gene et al. (1990); McKenzie and Jackson (1990); Ahmad et al. (1997); Pande and Rao (1998); Lumyong et al. (2003); Saravanan and Vittal (2007); Leão-Ferreira et al. (2010); Tarda et al. (2019)
* D. synnematicum* Somrith.	Decaying leaves of *Musa* sp.	India, Thailand	Somrithipol (2007)

A study was undertaken to determine the saprobic fungi associated with *Musa* sp. (banana) in Thailand, during the dry season. Three hyphomycetous taxa that morphologically resembled *Dictyoarthrinium* were examined. According to our phylogenetic analyses of combined SSU, LSU, ITS and *tef*1-α sequence data, *Dictyoarthrinium* clustered in Didymosphaeriaceae (Pleosporales, Dothideomycetes) with strong statistical support, sister to *Spegazzinia*. Hence, we propose to transfer *Dictyoarthrinium* from Apiosporaceae (Xylariales, Sordariomycetes) to Didymosphaeriaceae (Pleosporales, Dothideomycetes) and introduce *Dictyoarthrinium musae* sp. nov. as a saprobe recorded from *Musa* sp. We also provide detailed morphological illustrations, descriptions and DNA sequence data for *D. sacchari*, recorded on *Musa* sp. from Thailand, which further validates the novel taxonomic placement of *Dictyoarthrinium* in Didymosphaeriaceae.

## Materials and methods

### Sample collection, morphological studies and isolation

Dead leaves of *Musa* sp. were collected from Thailand during the dry season (December to August) of 2018 and 2019. Specimens were transferred to the laboratory in cardboard boxes. Samples were examined with a Motic SMZ 168 Series microscope. Powder-like masses of fungal conidia were mounted in water for microscopic studies and photomicrography. The specimens were examined using a Nikon ECLIPSE 80i compound microscope and photographed with a Canon 550D digital camera fitted to the microscope. Measurements were made with the Tarosoft (R) Image Frame Work programme and images used for figures were processed with Adobe Photoshop CS3 Extended v. 10.0 software (Adobe Systems, USA).

Single spore isolation was carried out following the method described in Chomnunti et al. (2014). Germinated spores were individually transferred to potato dextrose agar (PDA) plates and incubated at 25 °C in daylight. Colony characteristics were observed and measured after 3 weeks at 25 °C. Herbarium specimens were deposited in the Mae Fah Luang University (MFLU) Herbarium, Chiang Rai, Thailand. Living cultures were deposited in the Culture Collection of Mae Fah Luang University (MFLUCC). Faces of fungi numbers (Jayasiri et al. 2015) and MycoBank numbers (http://www.MycoBank.org) were obtained for the respective taxa.

### DNA extraction, PCR amplification and sequencing

Fungal isolates grown on potato dextrose agar (PDA) for 4 weeks at 25 °C were used to extract total genomic DNA. DNA was extracted from 50 to 100 mg of axenic mycelium of the 4-weeks-old growing cultures. The mycelium was ground to a fine powder in liquid nitrogen and fungal DNA was extracted using the Biospin Fungus Genomic DNA Extraction Kit-BSC14S1 (BioFlux, P.R. China) according to the manufacturer’s instructions. Four gene regions, the internal transcribed spacer (ITS), partial 18S small sub unit (SSU), partial 28S large sub unit (LSU) and partial translation elongation factor 1-alpha gene (*tef*1-α) were amplified using ITS5/ITS4 (White et al. 1990), NS1/NS4 (White et al. 1990), LR0R/LR5 (Vilgalys and Hester 1990) and EF1-983F /EF1-2218R (Rehner 2001) primers, respectively.

Polymerase chain reactions (PCR) were conducted according to the following protocol. The total volume of the PCR reaction was 25 μl and consisted of 12.5 μl of 2 × Power Taq PCR MasterMix (a premix and ready to use solution, including 0.1 Units/μlTaq DNA Polymerase, 500 μm dNTP Mixture each (dATP, dCTP, dGTP, dTTP), 20 mM Tris-HCl pH 8.3, 100 mMKCl, 3 mM MgCl_2_, stabiliser and enhancer), 1 μl of each primer (10 pM), 2 μl genomic DNA extract and 8.5 μl double distilled water (ddH_2_O). The reaction was conducted by running for 40 cycles. The annealing temperature was 56 °C for ITS and LSU, 57.2 °C for *tef*1-α and 55 °C for SSU and initially 95 °C for 3 min, denaturation at 95 °C for 30 seconds, annealing for 1 min, elongation at 72 °C for 30 seconds and final extension at 72 °C for 10 min for all gene regions. PCR amplification was confirmed on 1% agarose electrophoresis gels stained with ethidium bromide. The amplified PCR fragments were sent to a commercial sequencing provider (TsingKe Biological Technology Co., Beijing, China). The nucleotide sequence data acquired were deposited in GenBank.

### Sequence alignment

Sequences obtained in this study were subjected to BLAST search in GenBank (https://blast.ncbi.nlm.nih.gov/Blast.cgi). BLAST search results and initial morphological studies supported that our isolates belong to Didymosphaeriaceae. Other sequences used in the analyses were obtained from GenBank based on recently published papers (Tanaka et al. 2015; Jayasiri et al. 2019) (Table 2) and BLAST search results. The single gene alignments were done by MAFFT v. 7.036 (http://mafft.cbrc.jp/alignment/server/large.html; Katoh et al. 2019) using the default settings and later refined, where necessary, using BioEdit v. 7.0.5.2 (Hall 1999).

**Table 2.** Selected taxa with their corresponding GenBank accession numbers in the family Didymosphaeriaceae that are used in the phylogenetic analyses. Type strains are indicated as superscript T and newly-generated strains are indicated in bold.

Taxa	Culture collection	ITS	LSU	SSU	*tef*1-α
* Alloconiothyrium aptrootii*	CBS 980.95^T^	JX496121	JX496234	NA	NA
* A. aptrootii*	CBS 981.95^T^	JX496122	JX496235	NA	NA
* Austropleospora archidendri*	CBS 168.77^T^	JX496049	JX496162	NA	NA
* A. keteleeriae*	MFLUCC 18-1551^T^	NR_163349	MK348021	MK347910	MK360045
* Bambusistroma didymosporum*	MFLU 15-0057^T^	KP761733	KP761730	KP761737	KP761727
* B. didymosporum*	MFLU 15-0058	KP761734	KP761731	KP761738	KP761728
* Bimuria novae zelandiae*	CBS 107.79^T^	MH861181	AY016356	AY016338	DQ471087
* Chromolaenicola lampangensis*	MFLUCC 17-1462^T^	MN325016	MN325004	MN325010	MN335649
* C. thailandensis*	MFLUCC 17-1510^T^	MN325018	MN325006	MN325012	MN335651
* Cylindroaseptospora leucaenae*	MFLUCC 17-2424^T^	NR_163333	NG_066310	MK347856	MK360047
* Deniquelata barringtoniae*	MFLUCC 11-0422^T^	NR_111779	NG_042696	JX254656	NA
* D. vittalii*	NFCCI4249^T^	MF406218	MF182395	MF622059	MF182398
*** Dictyoarthrinium musae***	**MFLUCC 20-0105^T^**	**MT482323**	**MT482320**	**MT482326**	**MT495602**
*** D. musae***	**MFLUCC 20-0106^T^**	**MT482324**	**MT482321**	**MT482327**	**MT495603**
*** D. sacchari***	**MFLUCC 20-0107**	**MT482325**	**MT482322**	**MT482328**	NA
* D. sacchari*	CBS 529.73	NA	MH872479	NA	NA
* Didymocrea sadasivanii*	CBS 438.65^T^	MH858658	DQ384103	NA	NA
* Didymosphaeria rubi-ulmifolii*	MFLUCC 14-0023^T^	NA	KJ436586	NG_063557	NA
* D. rubi-ulmifolii*	MFLUCC 14-0024	NA	KJ436585	KJ436587	NA
* Kalmusia italica*	MFLUCC 14-0560^T^	KP325440	KP325441	KP325442	NA
* K. variisporum*	CBS 121.517^T^	NR_145165	JX496143	NA	NA
* Kalmusibambusa triseptata*	MFLUCC 13-0232^T^	KY682697	KY682695	KY682696	NA
* Karstenula rhodostoma*	CBS 690.94	NA	GU301821	GU296154	GU349067
* K. rhodostoma*	CBS 691.94	LC014559	AB807531	AB797241	AB808506
* Laburnicola hawksworthii*	MFLUCC 13-0602^T^	KU743194	KU743195	KU743196	NA
* L. muriformis*	MFLUCC 14-0921^T^	KU743200	KU743201	KU743202	NA
* Letendraea cordylinicola*	MFLUCC 11-0150	KM213996	KM213999	KM214002	NA
* L. cordylinicola*	MFLUCC 11-0148^T^	NR_154118	NG_059530	KM214001	NA
* Montagnula bellevaliae*	MFLUCC 14-0924^T^	KT443906	KT443902	KT443904	KX949743
* M. cirsii*	MFLUCC 13-0680	KX274242	KX274249	KX274255	KX284707
* M. scabiosae*	MFLUCC 14-0954^T^	KT443907	KT443903	KT443905	NA
* Neokalmusia brevispora*	KT 1466^T^	LC014573	AB524600	AB524459	AB539112
* N. scabrispora*	KT 1023	LC014575	AB524593	AB524452	AB539106
* Neptunomyces aureus*	CMG12^T^	MK912121	NA	NA	MK948000
* N. aureus*	CMG13	MK912122	NA	NA	MK948001
* Paracamarosporium fagi*	CPC 24890	KR611886	KR611904	NA	NA
* P. fagi*	CPC 24892^T^	KR611887	KR611905	NA	NA
* Paraconiothyrium cyclothyrioides*	CBS 972.95^T^	JX496119	JX496232	AY642524	NA
* Paramassariosphaeria anthostomoides*	CBS 615.86	MH862005	GU205223	GU205246	NA
* P. anthostomoides*	MFLU 16-0172^T^	KU743206	KU743207	KU743208	NA
* Paraphaeosphaeria rosae*	MFLUCC 17-2549^T^	MG828937	MG829046	MG829152	MG829223
* P. rosicola*	MFLUCC 15-0042^T^	NR_157528	MG829047	MG829153	NA
* Phaeodothis winteri*	CBS 182.58	NA	GU301857	GU296183	NA
* Pseudocamarosporium propinquum*	MFLUCC 13-0544	KJ747049	KJ813280	KJ819949	NA
* P. pteleae*	MFLUCC 17-0724^T^	NR_157536	MG829061	MG829166	MG829233
* Pseudopithomyces entadae*	MFLUCC 17-0917^T^	NA	NG_066305	MK347835	MK360083
* P. rosae*	MFLUCC 15-0035^T^	MG828953	MG829064	MG829168	NA
* Spegazzinia bromeliacearum*	URM 8084^T^	MK804501	MK809513	NA	NA
* S. deightonii*	MFLUCC 20-0002	MN956768	MN956772	MN956770	NA
* S. intermedia*	CBS 249.89^T^	MH862171	MH873861	NA	NA
* S. lobulata*	CBS 361.58^T^	MH857812	MH869344	NA	NA
* S. musae*	MFLUCC 20-0001^T^	MN930512	MN930514	MN930513	NA
* S. neosundara*	MFLUCC 15-0456^T^	KX965728	KX954397	KX986341	NA
* S. radermacherae*	MFLUCC 17-2285^T^	MK347740	MK347957	MK347848	MK360088
* S. tessarthra*	SH 287	JQ673429	AB807584	AB797294	AB808560
* Tremateia arundicola*	MFLU 16-1275^T^	KX274241	KX274248	KX274254	KX284706
* T. guiyangensis*	GZAAS01^T^	KX274240	KX274247	KX274253	KX284705
* T. murispora*	GZCC 18-2787^T^	NR_165916	MK972751	MK972750	MK986482
* Verrucoconiothyrium nitidae*	CBS:119209	EU552112	NA	NA	NA
* Xenocamarosporium acaciae*	CBS:139895	NR_137982	NG_058163	NA	NA
* X. acaciae*	MFLUCC 17-2432	MK347766	MK347983	MK347873	MK360093

*Abbreviations of culture collections: **CBS**: Centraalbureau voor Schimmelcultures, Utrecht, The Netherlands, **CPC**: Working collection of Pedro Crous housed at CBS, **GZAAS**: Guizhou Academy of Agricultural Sciences Herbarium, China, **KT**: K. Tanaka, **MFLU**: Mae Fah Luang University, Chiang Rai, Thailand, **MFLUCC**: Mae Fah Luang University Culture Collection, Chiang Rai, Thailand, **SH**: Academia Sinica People’s Republic of China. Shanghai, **URM**: Universidade Federal de Pernambuco.

### Phylogenetic analyses

**Figure 1.**
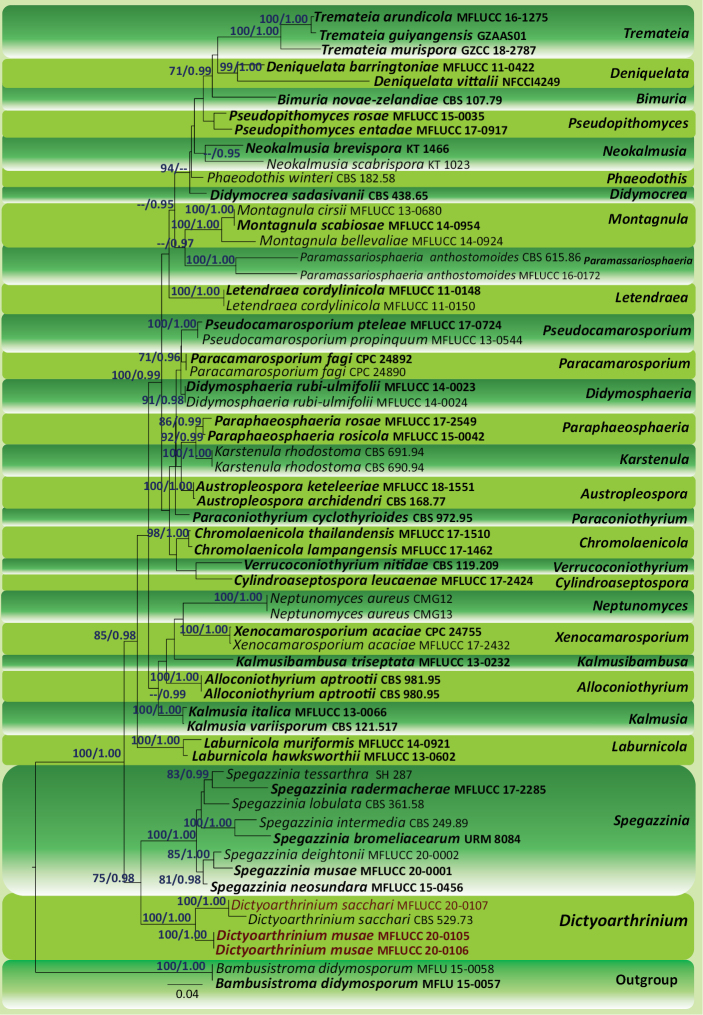
Maximum Likelihood tree revealed by RAxML from an analysis of SSU, LSU and ITS and *tef*1-α sequence data of the genera of Didymosphaeriaceae, showing the phylogenetic position of *Dictyoarthrinium musae* (MFLUCC 20-0105, MFLUCC 20-0106) and *D. sacchari* (MFLUCC 20-0107). ML bootstrap supports (≥ 60%) and Bayesian posterior probabilities (≥ 0.95 BYPP) are given above the branches, respectively. The tree is rooted with *Bambusistroma didymosporum* (MFLU 15-0057 and MFLU 15-0058). Strains generated in this study are indicated in brown bold type. Ex-type strains are indicated in black bold. The scale bar represents the expected number of nucleotide substitutions per site.

Maximum Likelihood (ML) trees were generated using the RAxML-HPC2 on XSEDE (8.2.8) (Stamatakis et al. 2008; Stamatakis 2014) in the CIPRES Science Gateway platform (Miller et al. 2010) using GTR+I+G model of evolution. Bootstrap supports were obtained by running 1000 pseudo-replicates. Maximum Likelihood bootstrap values (ML) ≥ 60% are given above each node of the phylogenetic tree in blue (Fig. 1).

Bayesian analysis was conducted with MrBayes v. 3.1.2 (Huelsenbeck and Ronquist 2001) to evaluate posterior probabilities (PP) (Rannala and Yang 1996; Zhaxybayeva and Gogarten 2002) by Markov Chain Monte Carlo sampling (BMCMC). Two parallel runs were conducted, using the default settings, but with the following adjustments: four simultaneous Markov chains were run for 2,000,000 generations, trees were sampled every 100^th^ generation and 20,001 trees were obtained. The first 4,000 trees, representing the burn-in phase of the analyses, were discarded. The remaining 16,001 trees were used for calculating PP in the majority rule consensus tree. Branches with Bayesian posterior probabilities (BYPP) ≥ 0.95 are indicated above each node of the phylogenetic tree (Fig. 1). Phylogenetic trees were visualised with the FigTree v1.4.0 programme (Rambaut 2011).

## Results

### Phylogenetic analyses

The combined SSU, LSU, ITS and *tef*1-α matrix comprised 61 sequences that represents the genera in Didymosphaeriaceae. The best scoring RAxML tree is shown (Fig. 1) with a final ML optimisation likelihood value of -19278.64. The matrix had 1091 distinct alignment patterns, with 39.08% of undetermined characters or gaps. Estimated base frequencies were: A = 0.234095, C = 0.252628, G = 0.278053, T = 0.235224; substitution rates AC = 1.252730, AG = 2.198875, AT = 1.318760, CG = 0.953798, CT = 5.276095, GT = 1.000000; proportion of invariable sites I = 0.491333; gamma distribution shape parameter α = 0.446418. All trees (ML and BYPP) were similar in topology and did not differ at the generic relationships, which are in agreement with multi-gene phylogeny of Tanaka et al. (2015) and Jayasiri et al. (2019). All *Dictyoarthrinium* strains analysed herein clustered as a highly-supported monophyletic clade (ML = 100%, BYPP = 1.00) in Didymosphaeriaceae (Fig. 1) sister to *Spegazzinia* (ML = 75%, BYPP = 0.98). We have included LSU sequence data of *D. sacchari* (CBS 529.73) of Vu et al. (2019) in our phylogenetic analyses. According to GenBank, CBS 529.73 was classified in Apiosporaceae (Sordariomycetes). In our analyses, *D. sacchari* (CBS 529.73) clustered with MFLUCC 20-0105, MFLUCC 20-0106 and MFLUCC 20-0107 strains in Didymosphaeriaceae with a strong statistical support (ML = 100%, BYPP = 1.00). Our strain MFLUCC 20-0107 grouped with *D. sacchari* (CBS 529.73). The novel isolates of *D. musae* (MFLUCC 20-0105 and MFLUCC 20-0106) were sister to *D. sacchari* (CBS 529.73 and MFLUCC 20-0107) with strong statistical support (ML = 100%, BYPP = 1.00).

## Taxonomy

### Dictyoarthrinium musae

Taxon classificationFungiPleosporales

Samarakoon, Chomnunti & K.D. Hyde sp. nov.

7BDFC66F-9636-52E8-8C6F-AC016DFBC029

MycoBank No: 835764

Facesoffungi Number: FoF08467

Figure 2

#### Etymology.

Name reflects the host genus, *Musa* (Musaceae).

#### Holotype.

MFLU 20-0437

#### Description.

*Saprobic* on dead leaves of *Musa* sp. **Sexual morph**: Undetermined. **Asexual morph**: *Colonies* compact or effuse, black, often pulvinate. *Mycelium* superficial, a close network of branched and anastomosing hyphae. *Stromata* none. *Setae* and *hyphopodia* absent. *Conidiophores* 30–140 × 1–2 μm (x¯ 81.5 × 1.6 μm, n = 25), basauxic, arising usually singly from subspherical, subhyaline to light brown conidiophore mother cells, 4.5–4.8 × 4.3–4.5 μm (x̄ = 4.6 × 4.4 μm, n = 10), macronematous, mononematous, straight or flexuous, narrow, cylindrical, rough, subhyaline to pale brown, with thick brown or dark brown transverse septa that appear as stripes with distances of 6.3–5.8 μm at apex and 2.3–3 μm at base of the conidiophore. *Conidiogenous cells* 4.1–4.5 × 4.3–4.7 μm (x̄ = 4.4 × 4.5 μm, n = 10), blastic, integrated, terminal and intercalary, cylindrical, smooth, denticles absent, hyaline. *Conidia* 7–11.5 × 6.5–9 μm (x̄ = 8.7 × 7.9 μm, n = 40), solitary, dry, acropleurogenous, simple, square, rounded at the corners, 4-celled, spherical or subspherical, often flattened in one plane, pale to dark brown at maturity, verrucose, with light brown to dark brown warts, immature conidia often 1-celled and subhyaline. Terminal conidium with four cells, sometimes absent or fallen before lateral conidia, mature conidia split along one line of the septa, most conidia arranged obliquely downwards on the conidiophore, conidial formation observed as a bunch starting after conidiophore 1–3 septate.

#### Culture characteristics.

Conidia germinating on PDA within 18 hrs. Colonies on PDA reaching a diameter of 50 mm after 14 days at 25 °C, slightly raised, hairy, filamentous, moderately dense, middle light grey, periphery white; reverse white to greyish-white.

#### Material examined.

THAILAND. Chiang Rai. On dead leaves of *Musa* sp. (Musaceae), 7 December 2018, M. C. Samarakoon, BNS265 (MFLU 20-0437, ***holotype***), ex-type living culture (MFLUCC 20-0105); *ibid*. 20 February 2019, B. C. Samarakoon BNS2239 (MFLU 20-0438, ***paratype***), ex-paratype living culture (MFLUCC 20-0106).

#### Notes.

Based on BLAST search results of SSU, LSU, ITS and *tef*1-α sequence data, *Dictyoarthrinium musae* (MFLUCC 20-0105 and MFLUCC 20-0106) showed high similarity as follows: SSU = 99.15% to *Paraconiothyrium hawaiiense* (CBS 120025), LSU = 95.57% to *Cylindroaseptospora siamensis* (MFLUCC 17-2527), ITS = 98.24% to *Kalmusia italica* (isolate 5), *tef*1-α = 97.75% to *Spegazzinia neosundara* (MFLUCC 13-0211) with 100%, 100%, 87% and 99% query covers, respectively. In the multigene phylogeny, the *Dictyoarthrinium* clade was sister to *Spegazzinia* (ML = 75%, BYPP = 0.98). Within the *Dictyoarthrinium* clade, *D. musae* (MFLUCC 20-0105 and MFLUCC 20-0106) separated from the sister taxon, *D. sacchari* with strong statistical support (ML = 100%, BYPP = 1.00). ITS sequence comparison revealed 7.84% base pair differences between *D. musae* and *D. sacchari* (MFLUCC 20-0107), which is in agreement with the new species concept outlined by Jeewon and Hyde (2016). *Dictyoarthrinium musae* differs from *D. sacchari* by its unique conidial development in the apex. The terminal conidia of *D. musae* are always 4-celled and similar in colour to mature lateral conidia. In addition, the terminal conidia of *D. musae* are sometimes absent or fallen before the lateral conidia. In contrast, the terminal conidia of *D. sacchari* can be 2-celled or 4-celled, pale brown with respect to lateral mature conidia and always persist on the conidiophore. In addition, the mature conidia of *D. musae* split along one line of the septa and this specific feature is absent in *D. sacchari*. *Dictyoarthrinium musae* has a subhyaline, spherical conidiophore mother cell while *D. sacchari* has a distinct cup-shaped, brown conidiophore mother cell. Therefore, based on contrasting morphological differences to *D. sacchari* and strong statistical support from our molecular phylogeny, *D. musae* is herein introduced as a new species.

**Figure 2.**
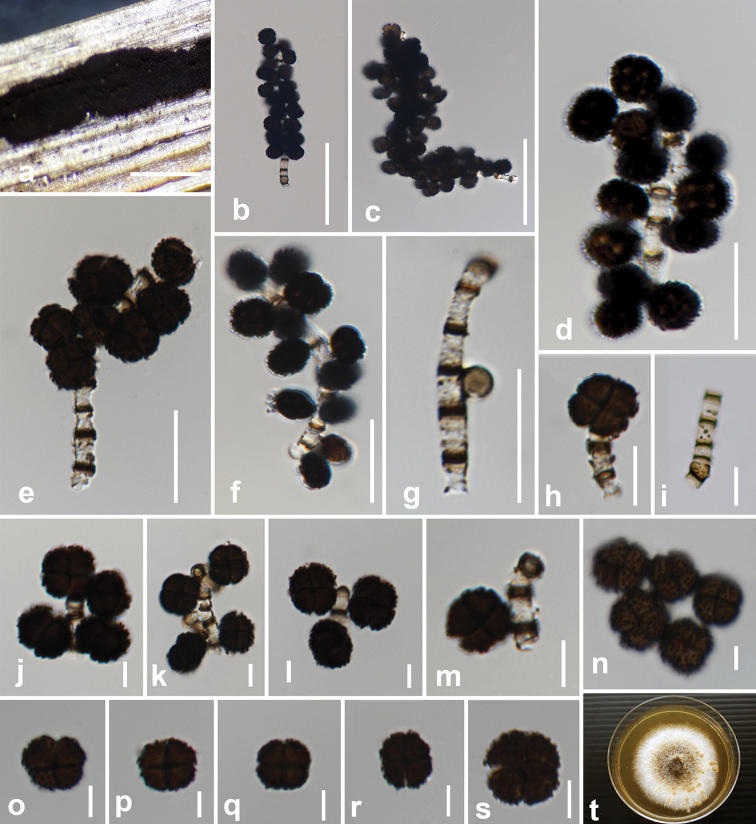
*
Dictyoarthrinium musae* (MFLU 20-0437, holotype) **a** conidia on the host **b** conidiophore and conidia with conidiophore mother cell **c–f** conidia with conidiophores on stalk **g** developmental stage of an immature lateral conidium **h** four-celled terminal conidium **i** conidiophore **j** conidiophores and conidia with terminal conidium **k, l** conidiophores without terminal conidium **m** attachment of a mature lateral conidium **n–q** warted four-celled mature conidia **r, s** mature conidia that split at septa **t** colony on PDA after 21 days. Scale bars: 500 μm (**a**); 50 μm (**b, c**); 20 μm (**d–g, i**); 10 μm (**h**); 5 μm (**j–s**).

### Dictyoarthrinium sacchari

Taxon classificationFungiPleosporales

(J.A. Stev.) Damon, Bull. Torrey bot. Club 80: 164 (1953)

639055B7-FFFC-5BE2-B06C-341C7E53270D

Facesoffungi Number: FoF08468

Figure 3

#### Description.

*Saprobic* on dead leaves of *Musa* sp. **Sexual morph**: Undetermined. **Asexual morph**: *Colonies* compact or effuse, black, often pulvinate. *Mycelium* superficial, a close network of branched and anastomosing hyphae. *Stromata* none. *Setae* and *hyphopodia* absent. *Conidiophores* 50–110 × 1–2 μm (x̄ = 72.0 × 1.6 μm, n = 15), basauxic, arising from cup-shaped, brown, distinct conidiophore mother cells, 3.4–4.4 × 2.9–4.7 μm (x̄ = 4 × 3.7 μm, n = 10), macronematous, mononematous, usually straight or flexuous, narrow, cylindrical, rough-walled, subhyaline to pale brown, with dark brown transverse septa as stripes with distances of 6.3–5.8 μm at apex and 2.3–3 μm at base of the conidiophore. *Conidiogenous cells* 4–4.5 × 4.3–4.7 μm (x̄ = 4.4 × 4.5 μm, n = 10), blastic, integrated, terminal and intercalary, cylindrical, smooth, hyaline. *Conidia* at maturity 8.5–11.5 × 8.5–10 μm (x̄ = 9.9 × 9.3 μm, n = 40), solitary, dry, acropleurogenous, simple, square, rounded at the corners, 4-celled, but difficult to distinguish the cells due to their blackish-brown nature, spherical or subspherical, often flattened in one plane, blackish-brown at maturity, with brown warts on surface of the cells, terminal conidium always 4-celled or 2-celled, light brown when compared with lateral conidia, most conidia arranged perpendicular to the conidiophore, some directed obliquely upwards.

#### Culture characteristics.

Conidia germinating on PDA within 18 hrs. Colonies on PDA reaching a diameter of 55 mm after 14 days at 25 °C, raised, moderately dense, entire margined, brownish-grey at maturity; reverse white to greyish-white.

#### Material examined.

Thailand, Chiang Mai. On mid-rib of a dead leaf of *Musa* sp. (Musaceae), S. Phongeun, 18 July 2018, BNS2287, (MFLU 20-0439), living culture MFLUCC 20-0107.

#### Notes.

Based on BLAST search results of SSU, LSU, ITS and *tef*1-α sequence data, our strain (MFLUCC 20-0107) showed high similarity to the taxa in GenBank as follows (SSU = 99.26% to *Paraconiothyrium brasiliense* (isolate GF1), LSU = 96.14% to *Alloconiothyrium aptrooti* (CBS 981.95), ITS = 93.00% to *Kalmusia italica* (MFLUCC 13-0066). In the multigene phylogeny, MFLUCC 20-0107 groups with *Dictyoarthrinium sacchari*, sister to *D. musae* with strong statistical support (ML = 100%, BYPP = 1.00). Our strain shares similar morphological features with *D. sacchari* (Subramanium 1952; Ellis 1971) and did not differ significantly. There are slight differences in conidial dimensions and the length of conidiophores of our collection and other *D. sacchari* collections by previous studies. Conidial dimensions and the length of conidiophores may differ due to diverse environmental effects and host associations. LSU sequence data of *D. sacchari* (CBS 529.73) are identical with our strain (MFLUCC 20-0107). Unfortunately, ITS, SSU and *tef*1-α sequence data of CBS 529.73 are not available in GenBank to compare with our strain. LSU data of *Dictyoarthrinium musae* have 2.24% of base pair difference with *D. sacchari* (CBS 529.73 and MFLUCC 20-0107). *Dictyoarthrinium sacchari* was reported on *Musa* sp. from Thailand in Lumyong et al. (2003) without morpho-molecular justifications. In this study, we document *D. sacchari* with detailed morphological illustrations, description, herbarium material and a living culture coupled with DNA sequence data (SSU, LSU, ITS) for a better taxonomic resolution.

**Figure 3.**
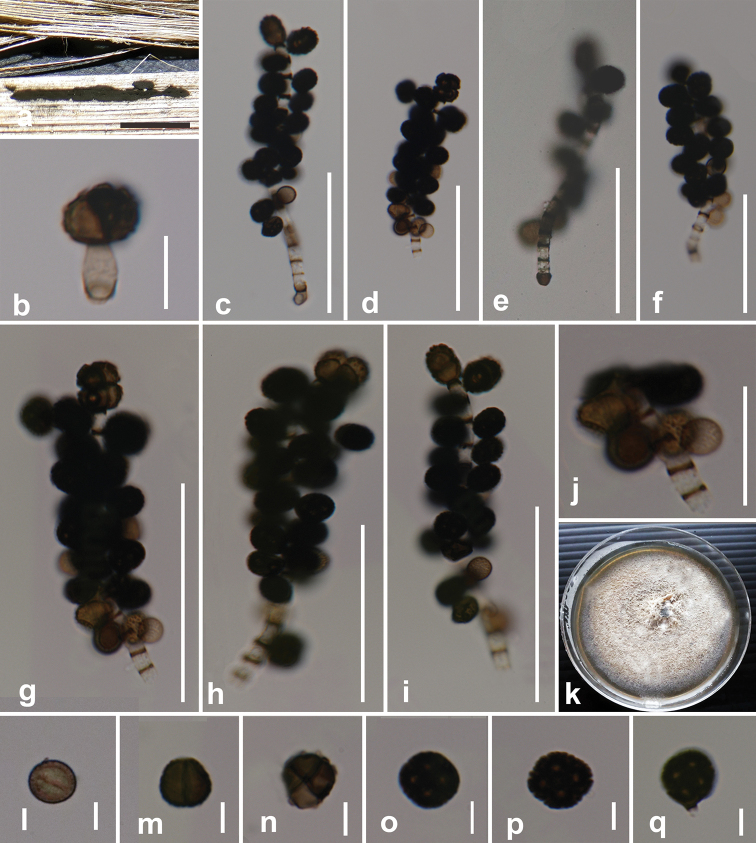
*
Dictyoarthrinium sacchari* (MFLU 20-0439) **a** conidia on the host **b** developmental stage of terminal conidium attached to the conidiophore **c–f** Conidiophores and conidia (**e**, with distinct mother cell) **g, h** mature conidiophores with four-celled terminal conidium **i** conidiophore with two celled terminal conidium **j** developmental stages of conidia on conidiophore **k** colony on PDA after 21 days **l–q** conidia. Scale bars: a = 1000 μm (**a**); 20 μm (**b, j**); 50 μm (**c–i**); 5 μm (**l–q**).

## Discussion

Both *Dictyoarthrinium* and *Spegazzinia* are characterised by basauxic conidiophores (Hughes 1952; Ellis 1971; Tanaka et al. 2015). *Spegazzinia* often has stellate (α) and disc-shaped (β) conidia (Ellis 1971; Tanaka et al. 2015). The conidia of *Dictyoarthrinium* (except *D. africanum*) share some similar characteristics with disc-shaped, β conidia of *Spegazzinia*. Both conidia are brown, 4-celled and constricted at the septa. Conidia of *Dictyoarthrinium* have characteristic hyaline or brown warts. Rarely, some taxa of *Spegazzinia*, for example, *S. deightonii*, also bear blunt ended spines. Most disc-shaped conidia of *Spegazzinia* are not warted. In addition, stellate conidia of *Spegazzinia* are always 4–5-celled and spinulose (Ellis 1971; Tanaka et al. 2015). There are contrasting morphological features of the basauxic conidiophores of both genera. The conidiophores of *Dictyoarthrinium* are hyaline to subhyaline with septa that appear as dark brown or light brown stripes throughout the conidiophore. The conidiophores (in stellate conidia) of *Spegazzinia* are more elongated, narrow, aseptate and dematiaceous.

*Dictyoarthrinium quadratum* (type of *Dictyoarthrinium*) is the heterotypic synonym of *D. sacchari. Dictyoarthrinium quadratum* has a terminal mature conidium with one to two cells. As described in Hughes (1952), these 2-celled conidia remain on the conidiophore, even when other conidia fall off. This feature is absent in *D. musae*. The terminal conidium of *D. musae* always ends up with four cells. The conidia of *D. quadratum* are obliquely upwardly directed, whereas the conidia of *D. musae* are obliquely downwardly directed (Fig. 2). The conidiophores of *D. quadratum* are erect and straight while *D. musae* has more curved conidiophores.

**Figure 4.**
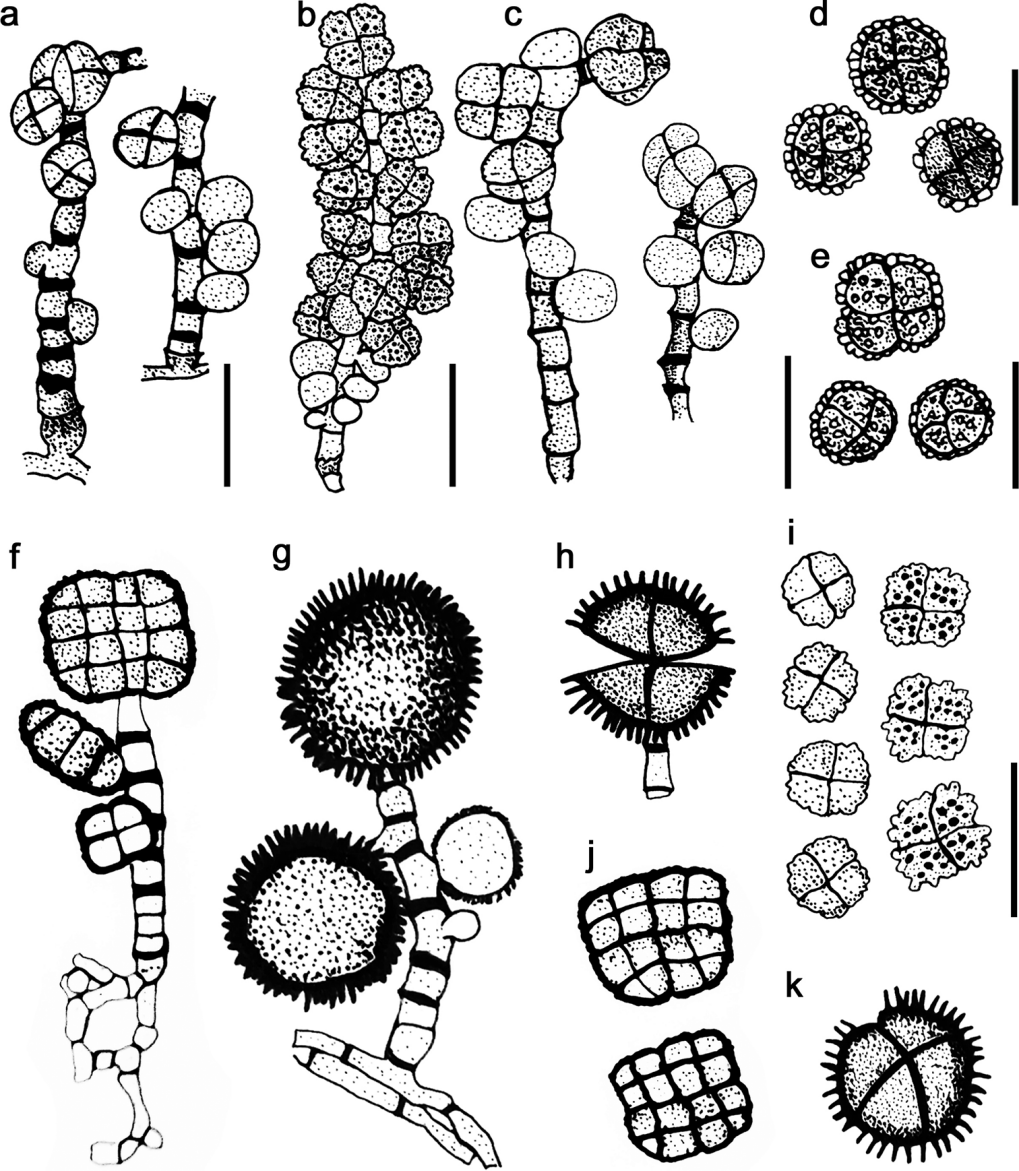
Morphology of conidia and conidiophores of previously described *Dictyoarthrinium* species **a, d***D. microsporum***b, i***D. synnematicum***c, e***D. lilliputeum***f, j***D. africanum***g, h, k***D. rabaulense*. Scale bars: 20 μm (**a, c, d, e**); 10 μm (**b, i**). Magnification × 650 (**f, g, h, j, k**). Redrawn from Rao and Rao (1964), Ellis (1971), Kobayasi et al. (1971) and Somrithipol (2007).

*Dictyoarthrinium africanum* differs significantly from *D. musae* by having 16-celled conidia. The conidia of *D. rabaulense* are completely black and densely echinulate with spines sometimes up to 4 μm long (Ellis 1976). However, *D. musae* has brown warts on the surface of conidia, while *D. lilliputeum* has hyaline warts. *Dictyoarthrinium microsporum* has longer conidiophores (250 μm) than *D. musae*. Morphological features of *Dictyoarthrinium* species are illustrated in Fig. 4. A key to the species of *Dictyoarthrinium* is provided below.

### Key to the species of *Dictyoarthrinium*

**Table:** 

1	Synnemata present	*** D. synnematicum***
–	Synnemata absent	**2**
2	Conidia 2- or 4-celled	**3**
–	Conidia 16-celled	*** D. africanum***
3	Conidia with brown warts	**4**
–	Conidia with hyaline warts	*** D. lilliputeum***
4	Conidiophores up to 130 μm long	**5**
–	Conidiophores up to 250 μm long	*** D. microsporum***
5	Terminal conidium always 4-celled, mature conidia split along one line of the septa ***D. musae***
–	Terminal conidium 2- or 4-celled, mature conidia do not split along septa	*** D. sacchari***

To date, the taxonomy and phylogeny of most genera that have basauxic conidiogenesis (Hughes 1952) have been resolved with their correct taxonomic placements. *Dictyoarthrinium* and *Endocalyx* represented the sole unresolved genera. We transferred *Dictyoarthrinium* to Didymosphaeriaceae based on morphological and molecular evidence. This study uses multigene sequence data of SSU, LSU, ITS and *tef*1-α for the first time to confirm the taxonomic placement of *Dictyoarthrinium* in Didymosphaeriaceae.

## Supplementary Material

XML Treatment for Dictyoarthrinium musae

XML Treatment for Dictyoarthrinium sacchari
